# Long-term oncological outcomes after oral cancer surgery using propofol-based total intravenous anesthesia versus sevoflurane-based inhalation anesthesia: A retrospective cohort study

**DOI:** 10.1371/journal.pone.0268473

**Published:** 2022-05-13

**Authors:** Lingju Miao, Xiang Lv, Can Huang, Ping Li, Yu Sun, Hong Jiang

**Affiliations:** Department of Anesthesiology, Shanghai Ninth People’s Hospital, Shanghai Jiao Tong University School of Medicine, Shanghai, China; University of Florida, UNITED STATES

## Abstract

**Background:**

Previous studies have shown that the anesthetic technique may influence long-term outcomes after cancer surgery. However, the association between the anesthetic technique and long-term oncological outcomes after oral cancer surgery remains unclear. Therefore, we conducted this study to address this gap.

**Methods:**

We reviewed the electronic medical records of patients who underwent elective oral cancer surgery between January 2014 and December 2015. The patients were grouped based on the anesthesia maintenance: either propofol or sevoflurane. Propensity score matching in a 1:1 ratio was performed to deal with the potential confounding effects of baseline characteristics. Univariate and multivariate Cox regression analyses were performed to compare hazard ratios (HRs) and identify the risk factors for death and recurrence. Survival analysis was performed using the Kaplan–Meier method, and survival curves were constructed from the date of surgery to death.

**Results:**

In total, 1347 patients were eligible for analysis, with 343 and 1004 patients in the propofol and sevoflurane groups, respectively. After propensity score matching, 302 patients remained in each group. Kaplan–Meier survival curves demonstrated the 5-year overall and recurrence-free survival rates of 59.3% and 56.0% and 62.7% and 56.5% in the propofol and sevoflurane groups, respectively. There was no significant difference in overall survival or recurrence-free survival between the groups. The multivariate Cox analysis verified this conclusion with HRs of 1.10 and 1.11 for overall survival and recurrence-free survival, respectively, in the sevoflurane group. Older age, advanced tumor-node-metastasis (TNM) stage, and American Society of Anesthesiologists class III were associated with poor overall survival. Patients with advanced TNM stage and poorly differentiated squamous cell carcinoma had a higher recurrence risk than their counterparts.

**Conclusion:**

The overall and recurrence-free survival rates were similar between propofol-based intravenous anesthesia and sevoflurane volatile anesthesia in patients who underwent oral cancer surgery.

## Introduction

Oral cancer is one of the most common malignancies, especially in developing and developed countries [1]. According to cancer statistics in China, approximately 48,100 patients were diagnosed with lip, oral cavity, and pharyngeal cancers, and 22,100 died in 2015 [2]. Surgical resection remains the gold standard treatment for curative purposes. However, in the perioperative period, both surgical stress and anesthesia may lead to tumor proliferation and metastasis [3]. Given that over 80% of patients with solid cancer will undergo at least one surgical procedure in an anesthesia state [4], it is necessary to determine the role of the anesthetic technique in the process of cancer recurrence.

Accumulating evidence has shown that anesthetics may influence tumor progression through immunomodulation effects [5, 6]. Intravenous anesthetics, mainly propofol, enhance antitumor immunity and therefore inhibit the invasion ability of cancer [7, 8]. In contrary, volatile anesthetics suppress immune function and have detrimental cancer-promoting effects [9–11]. In addition, data from experimental studies have shown that exposure to inhalation agents, but not propofol, enhances the malignancy of cancer by upregulating hypoxia-inducible factor-1ɑ (HIF-1ɑ) [12–14]. Such effects have also been found in clinical surgical settings [7, 15].

Although the data mentioned above suggest that propofol-based total intravenous anesthesia (TIVA) is superior to volatile anesthesia in cancer surgery, the results of some long-term survival studies are conflicting [16–22]. Some studies have reported that propofol-based TIVA is associated with better overall survival than inhalation anesthetics [16–18]. However, other studies did not show significant effects of the anesthetic technique on the prognosis of patients [19–22]. Recently, a meta-analysis including 19 retrospective studies involving patients undergoing breast, colon, gastroesophageal, glioma, hepatobiliary, and non-small cell lung cancer surgeries showed that propofol-based TIVA was associated with better overall survival than volatile anesthesia, especially in patients receiving desflurane inhalation [23]. However, to the best of our knowledge, patients with oral cancer have not yet been studied. Cancer aggressiveness and malignancy largely differ between cancer types and their origins [24]. In addition, oral cancer treatment usually includes radical tumor resection and immediate free flap tissue reconstruction, with an average operation time of more than 6 h. The effect of prolonged exposure to anesthesia on long-term oncological outcomes remains unknown. Therefore, we conducted a retrospective study to assess the association between the anesthetic technique and long-term oncological outcomes in patients after oral cancer surgery. We hypothesized that propofol-based TIVA would be associated with better long-term oncological outcomes after oral cancer surgery.

## Materials and methods

This retrospective cohort study was conducted at the Shanghai Ninth People’s Hospital, Shanghai Jiao Tong University School of Medicine. The Ethics Committee of Shanghai Ninth People’s Hospital approved this study and waived the need for informed consent on October 14, 2021 (approval number: SH9H-2021-T265-2).

### Participants and data sources

The electronic medical records of all patients who underwent curative oral cancer surgery at Shanghai Ninth People’s Hospital from January 2014 to December 2015 were reviewed. All relative data were extracted by department technicians who were blinded to the purpose of the study. The exclusion criteria were as follows: (1) American Society of Anesthesiologists (ASA) physical status greater than or equal to IV, (2) combined propofol anesthesia and volatile anesthesia, (3) missing medical records, (4) age less than 18 years, and (5) death within hospital stay.

### Anesthetic techniques

Patients were grouped according to the type of anesthesia they received, which was determined by the overall conditions of the patients and according to the preference of the attending anesthesiologists, which is usually the anesthesia at which they are most adept. No premedication was administered before anesthesia induction. General anesthesia was induced by midazolam (1–2 mg), fentanyl (1–2 μg/kg), propofol (1–2 mg/kg), and rocuronium (0.6–1 mg/kg) in all patients. Subsequently, nasal tracheal intubation was performed using a video-assisted laryngoscope or fiberoptic bronchoscope. Awake intubation was performed in patients with an estimated difficult airway or restricted mouth opening.

In the propofol-based TIVA group, anesthesia was maintained by target-controlled infusion (TCI, Fresenius Orchestra Primea; Fresenius Kabi AG, Germany) of propofol at an effect-site concentration of 2–6 μg/ml according to Schnider’s model. In the sevoflurane-based group, the vaporizer was adjusted between 1.5% and 5% according to the end-tidal concentration of sevoflurane. Remifentanil was continuously infused during the operation. The end-tidal carbon dioxide was maintained at 35–45 mmHg. The anesthesia depth was monitored using a bispectral index score (BIS) monitor (BIS VISTA monitoring System; Aspect Medical System, Inc., Norwood, MA, USA) within a range of 40–60. General care was consistent in both groups. After surgery, the patients were transferred to the post anesthesia care unit or intensive care unit for further observation, according to the anesthesiologist in charge. All patients in our institution received patient-controlled intravenous analgesia after surgery unless they refused or had other contraindications.

### Variables and outcome measurements

We retrospectively collected the following patient data from electronic medical records: patient demographics (sex, age, weight, height, ASA physical status, positive smoking, and alcoholism), tumor characteristics (anatomical site, tumor type, pathological tumor-node-metastasis stage [pTNM stage] according to the seventh edition of the Union for International Cancer Control guidelines), surgical and perioperative therapy (duration of anesthesia, reconstruction, transfusion data), and whether postoperative adjuvant radiotherapy or chemotherapy was provided. The age-adjusted Charlson comorbidity index was used to assess preoperative comorbidities. The Clavien–Dindo classification was used to assess postoperative surgical complications. The status of all patients was ascertained within 6 months before the closing date of the study (May 31, 2021).

The primary endpoint was overall survival, which was defined as the date of surgery to the date of death. The secondary endpoint was recurrence-free survival, defined as the date of surgery to the date of recurrence. The date of recurrence was the date on which the patient was diagnosed with recurrence during the outpatient follow-up visit. Survival time was censored in patients who were lost to follow-up before the closing date or in patients who were withdrawn at the closing date.

### Statistics analyses

According to the literature, the 5-year survival rate of oral cancer is normally between 50% and 60% [25, 26]. Assuming a 20% reduction in mortality with propofol anesthesia, and to achieve a power of 90% and a two-tailed type I error of α = 0.05, 127 patients were needed in each matched group.

For continuous variables, Student’s t-test was used to analyze log-normal distribution; otherwise, the Mann–Whitney U test was used. The chi-squared test was used to compare the categorical variables. The Kaplan–Meier method was used to analyze the overall survival and recurrence-free survival of patients from the date of surgery to the date of events. A univariate Cox regression analysis was performed, and then variables were included in the multivariate Cox regression model to identify the risk factors for death and recurrence if the p-value was less than 0.1. Propensity score matching was performed to account for the potential confounding effects of baseline characteristics and to compensate for the difference in the number of patients between the groups. The variables used for matching were as follows: sex, age, body mass index (BMI), ASA physical status, age-adjusted Charlson comorbidity score, positive smoking, alcoholism, anatomical site, tumor type, pTNM stage, duration of anesthesia, transfusion, reconstruction, postoperative adjuvant radiotherapy, or chemotherapy. Patients were matched at a 1:1 ratio using the nearest neighbor method with a caliper of width equal to 0.2 standard deviation of the logit of the propensity score. The standardized mean difference (SMD) for variables less than 0.1 were recognized as a good balance.

All analyses were performed using IBM SPSS Statistics version 26.0 (IBM Corp., Armonk, N.Y., USA), and Stata/IC 15.0 (Stata Corp, USA) was used to draw Kaplan–Meier curves. A two-tailed *p* value <0.05 was considered significant for all tests.

## Results

**Fig 1:**
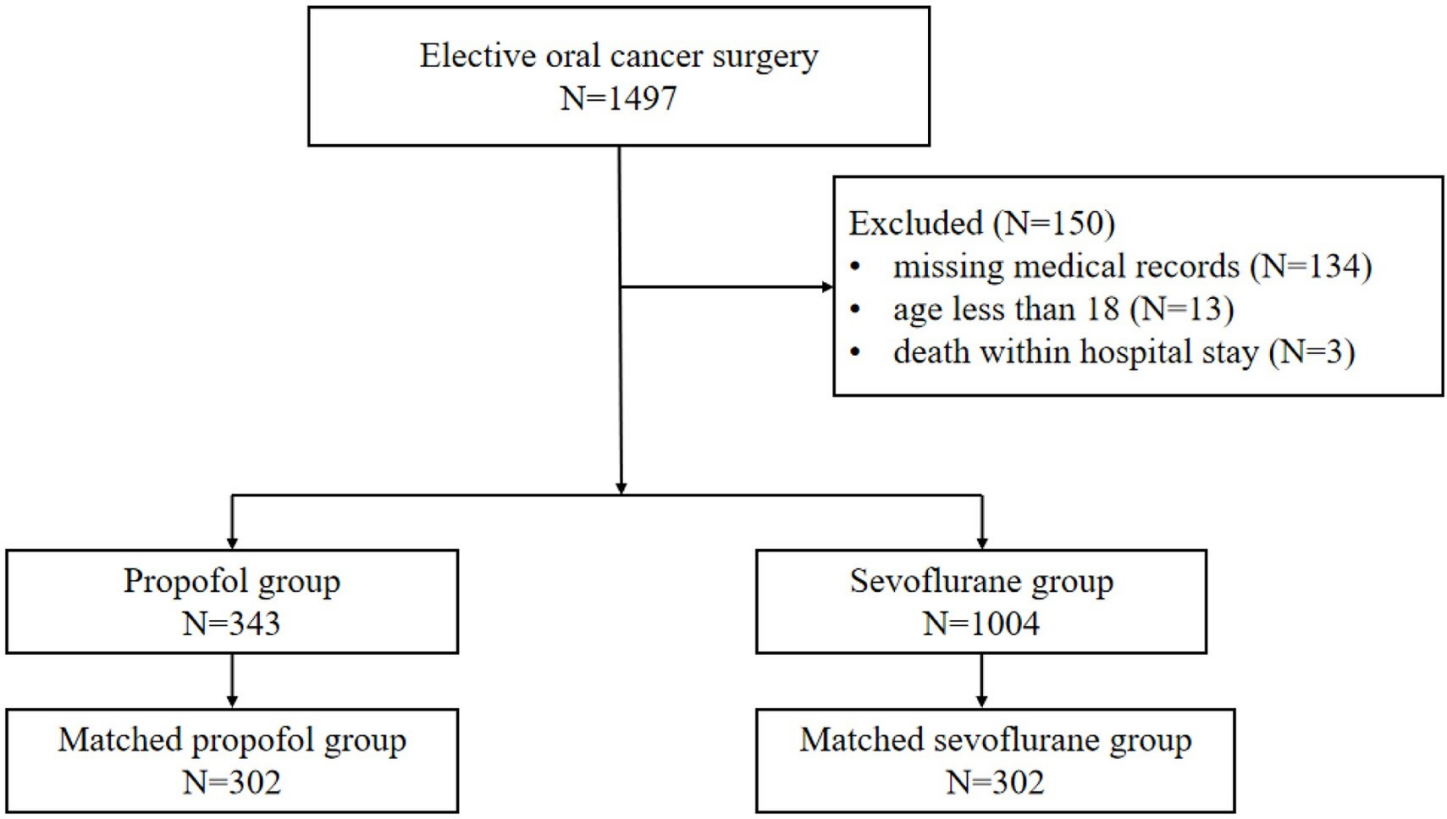
Flow diagram of the study population.

A total of 1497 patients were diagnosed with oral cancer and underwent curative surgery during the study period. After exclusions were applied, 1347 patients were included in the analysis, with 343 and 1004 patients in the propofol-based TIVA and sevoflurane-based groups, respectively (Fig 1). Compared with the sevoflurane group, patients in the propofol group were more likely to have a grade of ASA III and higher age-adjusted Charlson comorbidity index. The tongue was the most common tumor site in both groups, especially in the propofol group. After propensity score matching, all variables in each group were well balanced (p>0.05 and SMD<0.1). The patients and treatment characteristics for the total cohort and propensity-matched cohort are shown in Table 1.

**Table 1:** Patients and treatment characteristics for the overall group and the matched group.

Variables	Overall patients			Matched patients			
	Propofol (n = 343)	Sevoflurane (n = 1004)	p-value	Propofol (n = 302)	Sevoflurane (n = 302)	p-value	SMD
Age (yr)	58.4±12.8	58.4±12.6	0.981	57.3±12.8	57.6±13.1	0.802	0.021
Body mass index, kg/m^2^	22.3±3.3	22.7±3.6	0.119	22.3±3.4	22.2±3.8	0.741	0.027
Male sex	225 (65.6)	659 (65.6)	0.989	192 (63.6)	188 (62.3)	0.736	0.028
ASA physical status			<0.001			0.471	
• I	31 (9.0)	203 (20.2)		31 (10.3)	40 (13.2)		0.074
• II	257 (74.9)	784 (78.1)		256 (84.8)	245 (81.1)		0.088
• III	55 (16.0)	17 (1.7)		15 (5.0)	17 (5.6)		0.051
Age adjusted CCI	4.3±1.8	3.7±1.7	<0.001	4.1±1.7	4.1±1.7	0.778	0.023
Smoking	145 (42.3)	436 (43.4)	0.710	124 (41.1)	118 (39.1)	0.618	0.040
Alcoholism	105 (30.6)	279 (27.8)	0.317	87 (28.8)	77 (25.5)	0.360	0.074
pTNM stage			0.197			0.924	
• I	69 (20.1)	224 (22.3)		64 (21.2)	71 (23.5)		0.056
• II	103 (30.0)	249 (24.8)		91 (30.1)	88 (29.1)		0.023
• III	72 (21.0)	247 (24.6)		64 (21.2)	63 (20.9)		0.008
• IV	99 (28.9)	284 (28.3)		83 (27.5)	80 (26.5)		0.022
Anatomical site			<0.001			0.786	
• Tongue	159 (46.4)	348 (34.7)		140 (46.4)	133 (44.0)		0.049
• Buccal	55 (16.0)	156 (15.5)		46 (15.2)	56 (18.5)		0.091
• Gums	46 (13.4)	149 (14.8)		41 (13.6)	45 (14.9)		0.037
• Floor of mouth	50 (14.6)	132 (13.1)		42 (13.9)	37 (12.3)		0.049
• Others	33 (9.6)	219 (21.8)		33 (10.9)	31 (10.3)		0.016
Tumor type			0.110			0.769	
• Well differentiated SCC	39 (11.4)	145(14.4)		34 (11.3)	34 (11.3)		0.000
• Middle differentiated SCC	233 (67.9)	609 (60.7)		203 (67.2)	205 (67.9)		0.014
• Poor differentiated SCC	26 (7.6)	84 (8.4)		24 (7.9)	29 (9.6)		0.060
• Others	45(13.1)	166 (16.5)		41 (13.6)	34 (11.3)		0.062
Reconstruction	255 (74.3)	723 (72.0)	0.403	224 (74.2)	220 (72.8)	0.712	0.029
Anesthetic time, min	491.2±176.1	500.8±182.0	0.397	490.4±178.6	481.8±176.9	0.553	0.047
Transfusion	118 (34.4)	379 (37.7)	0.267	100 (33.1)	110 (36.4)	0.393	0.068
Clavien-Dindo classification			0.235			0.196	
• 0	204 (59.5)	644 (64.1)		183 (60.6)	197 (65.2)		
• I	38 (11.1)	115 (11.5)		32 (10.6)	38 (12.6)		
• II	66 (19.2)	148 (14.7)		59 (19.5)	40 (13.2)		
• III	35 (10.2)	97 (9.7)		28 (9.3)	27 (8.9)		
Radiotherapy	200 (58.3)	603 (60.1)	0.568	179 (59.3)	189 (62.6)	0.404	0.068
Chemotherapy	119 (34.7)	353 (35.2)	0.876	106 (35.1)	116 (37.4)	0.554	0.049
Recurrence	127 (37.0)	343 (34.2)	0.337	109 (36.1)	113 (37.4)	0.736	
All-cause mortality	128 (37.3)	304 (30.3)	0.016	109 (36.1)	105 (34.8)	0.734	

Data shown as mean ± SD or n (%). ASA: American Society of Anesthesiologists. CCI: Charlson comorbidity index. pTNM: pathological tumor-node-metastasis. SCC: squamous cell cancer. SMD: standardized mean difference.

**Fig 2:**
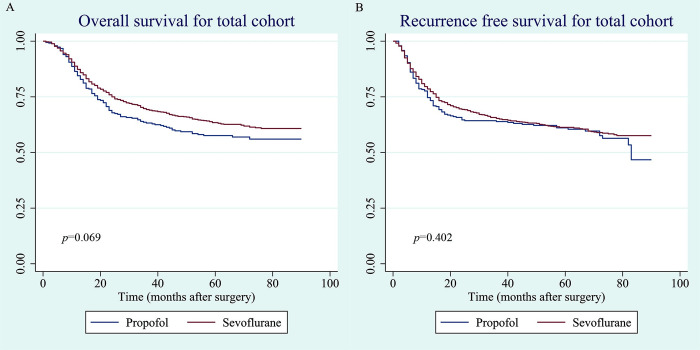
(A). Overall survival curve for total cohort. (B). Recurrence-free survival curve for total cohort.

In the total cohort, the median follow-up durations were 68 (interquartile range, 33–77), 62 (interquartile range, 43–73), and 69 (interquartile range, 24–77) months for all patients, the propofol group, and the sevoflurane group, respectively. During follow-up, the overall mortality rates were 32.1% (432/1347), 37.3% (128/343), and 30.3% (304/1004) in the entire cohort, the propofol group, and the sevoflurane group, respectively. The recurrence rates were 34.9% (470/1347), 37.0% (127/343), and 34.2% (343/1004) in all patients, the propofol group, and the sevoflurane group, respectively. Based on the Kaplan–Meier curves, the 1-, 3-, and 5-year overall survival rates were 84.5 (95% CI, 80.0–88.0), 63.2% (95% CI, 57.5–68.4), and 57.6% (95% CI, 51.7–63.1) and 87.3 (95% CI, 84.9–89.4), 69.3% (95% CI, 66.0–72.4), and 63.4% (95% CI, 59.9–66.7) in the propofol and sevoflurane groups, respectively. The Kaplan–Meier curves for overall survival (p = 0.069) and recurrence-free survival (p = 0.402) in the total cohort are shown in Fig 2.

**Fig 3:**
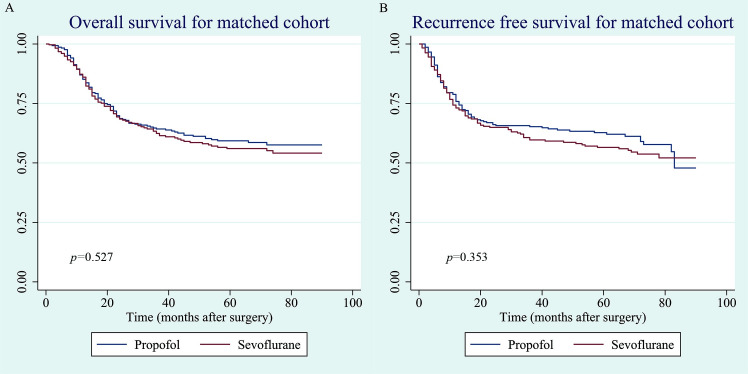
(A). Overall survival curve for matched cohort. (B). Recurrence-free survival curve for matched cohort.

In the propensity-matched cohort, based on the Kaplan–Meier survival curves, the 1-, 3-, and 5-year overall survival rates were 85.2% (95% CI, 80.4–88.8), 64.3% (95% CI, 58.2–69.7%), and 59.3% (95% CI, 53.0–65.0) and 86.1% (95% CI, 81.2–90.0), 62.4% (55.9–68.3%), and 56.0% (95% CI, 49.3–62.2%) in the propofol and sevoflurane groups, respectively (Fig 3). The 1-, 3-, and 5-year recurrence free survival rates were 75.8% (95% CI, 70.4–80.4), 65.2% (95% CI, 59.3–70.6), and 62.7% (95% CI, 56.6–68.3) and 73.1% (95% CI, 67.3–78.0), 59.7% (95% CI, 53.2–65.6), and 56.5% (95% CI, 49.9–62.6) in the propofol and sevoflurane groups, respectively (Fig 3). There was no significant difference in overall survival (p = 0.527) or recurrence-free survival (p = 0.353) between the propofol and sevoflurane groups (Fig 3).

In Tables 2 and 3, univariate and multivariate Cox regression analyses were performed to identify the risk factors for death and recurrence in the propensity-matched cohort. In the univariate analysis of overall survival, 11 factors with a p value <0.1 were included in the multivariate model (Table 2). After multivariate analysis, no significant difference was found for overall survival in the sevoflurane group (hazard ratio [HR] = 1.10; 95% CI, 0.84–1.45; p = 0.500 [Table 2]) as compared to the propofol group. Older age (HR = 1.04; 95% CI, 1.02–1.05; p<0.001), advanced TNM stage (TNM III: HR = 1.78; 95% CI, 1.11–2.86; p = 0.016 and TNM IV: HR = 2.03; 95% CI, 1.26–3.27; p = 0.004), and ASA class III (HR = 2.26; 95% CI, 1.02–5.00; p = 0.044) were associated with poor overall survival (Table 2). The results also showed that a high BMI was associated with a reduced risk of death (HR = 0.95; 95% CI, 0.92–0.99; p = 0.024). In the multivariate analysis of recurrence, the recurrence risk for patients in the propofol group was as maintained as that patients in the sevoflurane group (HR = 1.11; 95% CI, 0.85–1.45; p = 0.439; Table 3). Patients with advanced TNM stage (TNM III: HR = 1.57; 95% CI, 1.00–2.48; p = 0.051 and TNM IV: HR = 1.86; 95% CI, 1.17–2.95; p = 0.008) and poorly differentiated squamous cell carcinoma (HR = 1.88; 95% CI, 1.03–3.46; p = 0.042) had a higher recurrence risk than their counterparts (Table 3).

**Table 2:** Univariate and multivariate Cox regression analyses for overall survival in the matched cohort.

Variables	Univariable		Multivariable	
	HR (95%CI)	p-value	HR (95%CI)	p-value
Anesthesia (ref: propofol)	1.09 (0.83–1.43)	0.530	1.10 (0.84–1.45)	0.500
Age (yr)	1.04 (1.03–1.05)	<0.001	1.04 (1.02–1.05)	<0.001
Body mass index, kg/m^2^	0.95 (0.92–0.99)	0.018	0.95 (0.92–0.99)	0.024
Male (ref: female)	1.16 (0.88–1.54)	0.289		
ASA physical status (ref: I)				
• II	2.58 (1.44–4.63)	0.001	1.60 (0.86–2.97)	0.136
• III	6.41 (3.18–12.89)	<0.001	2.26 (1.02–5.00)	0.044
Age adjusted CCI	1.24 (1.15–1.34)	<0.001	1.01 (0.90–1.13)	0.859
Smoking (ref: no)	1.15 (0.88–1.51)	0.305		
Alcoholism (ref: no)	1.13 (0.84–1.52)	0.404		
pTNM stage(ref: I)				
• II	1.25 (0.81–1.93)	0.310	1.11 (0.71–1.74)	0.635
• III	2.00 (1.31–3.06)	0.001	1.78 (1.11–2.86)	0.016
• IV	2.40 (1.59–3.62)	<0.001	2.03 (1.26–3.27)	0.004
Anatomical site (ref: tongue)				
• Buccal	1.35 (0.93–1.96)	0.120		
• Gums	1.10 (0.73–1.66)	0.646		
• Floor of mouth	1.26 (0.84–1.88)	0.260		
• Others	1.39 (0.89–2.17)	0.149		
Tumor type (ref: well differentiated SCC)				
• Middle differentiated SCC	1.63 (0.99–2.70)	0.055	1.21 (0.72–2.03)	0.468
• Poor differentiated SCC	2.35 (1.25–4.44)	0.008	1.75 (0.92–3.35)	0.091
• Others	1.31 (0.70–2.44)	0.396	1.52 (0.81–2.86)	0.196
Reconstruction (ref: no)	1.27 (0.92–1.75)	0.143		
Anesthetic time, min	1.00 (1.00–1.00)	0.012	1.00 (1.00–1.00)	0.803
Transfusion (ref: no)	1.66 (1.26–2.17)	<0.001	1.31 (0.95–1.80)	0.103
Calvien-Dindo classification (ref: 0)				
• I	1.09 (0.71–1.68)	0.701	1.13 (0.72–1.78)	0.601
• II	1.55 (1.10–2.19)	0.013	1.21 (0.84–1.75)	0.314
• III	1.31 (0.86–2.01)	0.209	0.83 (0.53–1.31)	0.425
Radiotherapy (ref: no)	1.36 (1.02–1.81)	0.033	1.17 (0.83–1.66)	0.376
Chemotherapy (ref: no)	1.30 (0.99–1.70)	0.060	1.17 (0.85–1.60)	0.327

ASA: American Society of Anesthesiologists. CCI: Charlson comorbidity index. pTNM: pathological tumor-node-metastasis. SCC: squamous cell cancer.

**Table 3:** Univariate and multivariate Cox regression analyses for recurrence-free survival in the matched cohort.

Variables	Univariable		Multivariable	
	HR (95%CI)	p-value	HR (95%CI)	p-value
Anesthesia (ref: propofol)	1.13 (0.87–1.47)	0.358	1.11 (0.85–1.45)	0.439
Age (yr)	1.01 (1.00–1.02)	0.067	1.01 (1.00–1.02)	0.130
Body mass index, kg/m^2^	0.96 (0.92–1.00)	0.031	0.97 (0.93–1.01)	0.093
Male (ref: female)	1.15 (0.87–1.52)	0.315		
ASA physical status (ref: I)				
• II	1.54 (0.97–2.44)	0.069	1.34 (0.82–2.20)	0.248
• III	2.50 (1.29–4.83)	0.006	1.68 (0.80–3.53)	0.168
Age adjusted CCI	1.04 (0.96–1.12)	0.369		
Smoking (ref: no)	1.05 (0.80–1.37)	0.733		
Alcoholism (ref: no)	0.94 (0.69–1.27)	0.679		
pTNM stage(ref: I)				
• II	1.12 (0.74–1.70)	0.588	1.04 (0.68–1.60)	0.854
• III	1.75 (1.16–2.65)	0.007	1.57 (1.00–2.48)	0.051
• IV	2.17 (1.47–3.21)	<0.001	1.86 (1.17–2.95)	0.008
Anatomical site (ref: tongue)				
• Buccal	1.12 (0.76–1.63)	0.571	1.03 (0.69–1.53)	0.899
• Gums	0.89 (0.59–1.36)	0.602	0.95 (0.62–1.46)	0.812
• Floor of mouth	1.15 (0.77–1.70)	0.505	1.07 (0.71–1.60)	0.762
• Others	1.47 (0.98–2.22)	0.065	1.57 (0.98–2.52)	0.062
Tumor type (ref: well differentiated SCC)				
• Middle differentiated SCC	1.41 (0.86–2.30)	0.173	1.00 (0.60–1.67)	>0.999
• Poor differentiated SCC	2.68 (1.49–4.84)	0.001	1.88 (1.03–3.46)	0.042
• Others	1.48 (0.82–2.65)	0.191	1.32 (0.71–2.48)	0.383
Reconstruction (ref: no)	1.37 (0.99–1.88)	0.056	0.97 (0.58–1.60)	0.893
Anesthetic time, min	1.00 (1.00–1.00)	0.002	1.00 (1.00–1.00)	8.836
Transfusion (ref: no)	1.63 (1.25–2.13)	<0.001	1.34 (0.98–1.82)	0.068
Calvien-Dindo classification (ref: 0)				
• I	1.09 (0.72–1.65)	0.691	1.05 (0.68–1.61)	0.843
• II	1.44 (1.02–2.02)	0.038	1.24 (0.86–1.80)	0.256
• III	0.94 (0.59–1.51)	0.806	0.75 (0.45–1.24)	0.262
Radiotherapy (ref: no)	1.54 (1.15–2.04)	0.003	1.32 (0.94–1.87)	0.111
Chemotherapy (ref: no)	1.32 (1.01–1.72)	0.040	1.06 (0.78–1.44)	0.712

ASA: American Society of Anesthesiologists. CCI: Charlson comorbidity index. pTNM: pathological tumor-node-metastasis. SCC: squamous cell cancer.

**Fig 4:**
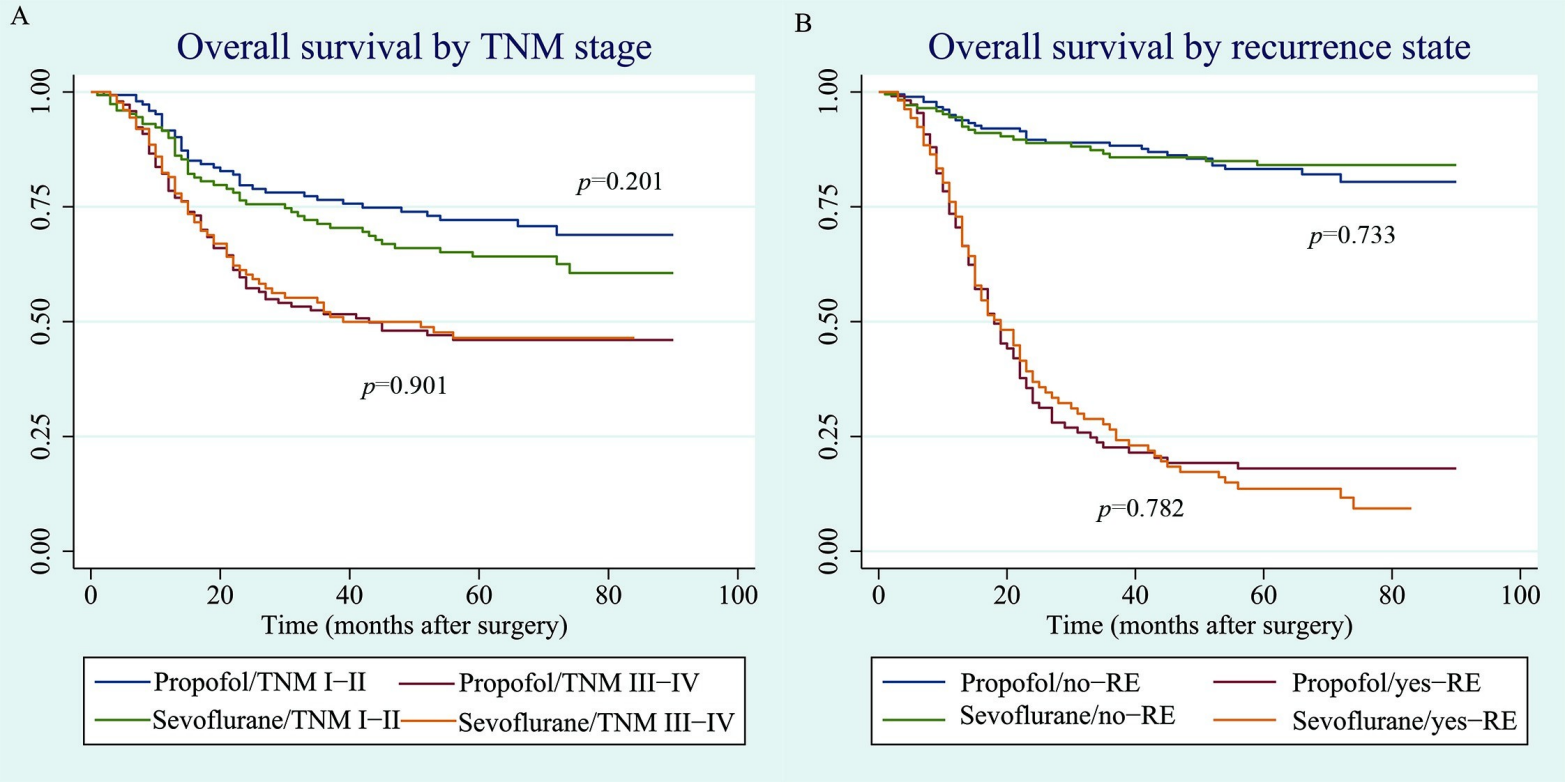
(A). Overall survival curve by TNM stage. TNM: tumor-node-metastasis. (B). Overall survival curve by recurrence state. RE: recurrence.

Subgroup analysis showed that the 5-year overall survival rates for TNM stages I–II were 72.1% (95% CI, 63.6–79.0) and 64.2% (95% CI, 55.0–72.0) in the propofol and sevoflurane groups, respectively (Fig 4). For TNM stages III–IV, the 5-year overall survival rates were 46.0% (95% CI, 37.1–54.5) and 46.5% (95% CI, 36.6–55.7) in the propofol and sevoflurane groups, respectively (Fig 4). The Kaplan–Meier curves suggested that regardless of TNM stage (TNM stages I–II, p = 0.201; TNM stages III–IV, p = 0.901), there was no difference in overall survival between the groups (Fig 4). In addition, subgroup analysis for recurrence state suggested that the overall survival for the propofol and sevoflurane groups was not significantly different in patients with (p = 0.782) or without (p = 0.733) recurrence (Fig 4).

## Discussion

Both propofol and sevoflurane are widely used for the maintenance of anesthesia in oral cancer surgery. General anesthetics may affect tumor cells and cell-mediated immunity [6]. The major finding of this retrospective study is that, for patients with oral cancer, the use of either propofol-based TIVA or sevoflurane-based inhalation anesthesia did not affect the overall survival. Our results also showed that propofol was not significantly associated with better recurrence-free survival by Cox regression analysis.

Currently, most of the studies have focused on anesthesia, and the long-term outcomes of patients receiving anesthesia are retrospectively analyzed, with inherent limitations. One meta-analysis of ten studies (one prospective randomized controlled trial [RCT] and nine retrospective studies) concluded that propofol-based TIVA was associated with improvements in recurrence-free and overall survival. After subgroup analysis, a significant effect was found for gastrointestinal malignancies [27]. The authors did not perform a subgroup analysis based on the inhaled anesthetics. The latest meta-analysis of 19 retrospective studies also suggested that propofol-based TIVA was associated with better overall survival [23]. After subgroup analysis, these studies demonstrated that propofol-based TIVA could provide better recurrence-free outcomes and overall survival in gastrointestinal cancer and hepatobiliary cancer surgeries. Furthermore, subgroup analysis by different volatile anesthetics showed that propofol-based TIVA was associated with better overall survival than desflurane rather than sevoflurane. Only one RCT comparing propofol-based TIVA versus sevoflurane anesthesia in breast cancer surgery did not find differences in 2-year recurrence-free and overall survival, although TIVA effectively inhibited the release of vascular endothelial growth factor (VEGF) [28]. Our results also confirmed that propofol-based TIVA could not provide benefits of long-term oncological outcomes compared with sevoflurane for patients with oral cancer. Taken together, the effect of general anesthetics may be dependent on a number of factors, including the type of tumor and type of anesthetic. Propofol-based TIVA is more likely to provide better long-term survival in gastrointestinal and hepatobiliary cancer surgeries than volatile anesthesia. Moreover, sevoflurane may be less likely to cause tumor metastasis than isoflurane and desflurane.

Several previous laboratory and clinical studies have provided several explanations. First, the effects of volatile anesthetics on tumor cells are conflicting. HIFs regulate a vast array of genes involved in tumor cell proliferation. Isoflurane was found to induce the upregulation of HIF-1α and enhancement of human renal cancer cell RCC4 [14] and prostate cancer cell PC3 [12] and ovarian cancer [29] proliferation and migration. Interestingly, sevoflurane, another structurally similar volatile anesthetic, is slightly different. Ferrell et al. first revealed that exposure to sevoflurane may increase the expression of pro-oncogenic protein markers in head and neck squamous cell carcinoma (HNSCC) cells by activating HIF-2α [30]. However, sevoflurane has also been reported to suppress hypoxia-induced growth and metastasis of lung cancer cells by inhibiting HIF-1α [31]. Our previous study found that sevoflurane inhibited the malignant potential of HNSCC by activating the HIF-1α signaling pathway [32]. We also found that sevoflurane attenuated hypoxia-induced VEGF levels in tongue squamous cell carcinoma cells by upregulating the DNA methylation status of the promoter region [33]. HIFs seemed to play a different role in the association between volatile anesthetics and tumor metastasis. In addition, sevoflurane has also been demonstrated to inhibit the invasion of cancer cells, such as colorectal [34], lung cancer [35] and breast cancer cells [36], through different molecular mechanisms. A random-controlled study suggested that desflurane and sevoflurane produced different stress responses during laparoscopic surgery, desflurane anesthesia was associated with a greater release of catecholamines [37] which has been suggested to facilitate metastatic processes [38, 39]. Second, propofol has been confirmed to induce slightly less effect on cellular immune responses than sevoflurane [11, 15, 40, 41]. However, propofol has different effects on tumor cells depending on the exposure time. For example, exposure for 24 h attenuated the invasion and migration of breast cancer cells MDA-MB-231 cells by inhibiting nuclear factor kappa B activity [42], whereas shorter exposure durations (less than 12 h) increased the proliferation and migration of MDA-MB-231 cells [43]. Third, surgical stress may be an important factor. Propofol is most likely to demonstrate benefits in patients requiring large surgical procedures that cause considerable tissue injury and provoke substantial neural and inflammatory responses [44]. In terms of activation of neural and inflammatory signaling pathways, oral cancer surgery is likely less invasive than surgeries such as laparotomy gastrointestinal tumor surgery. Certainly, these are assumptions and need to be clarified in further prospective RCTs.

Before propensity score matching, the all-cause mortality in the propofol group was significantly higher than that in the sevoflurane group. This may be attributed to the patients in the propofol group having higher proportion of ASA class III and higher age-adjusted Charlson comorbidity index. In our study, the age-adjusted Charlson comorbidity index seems to have no association with overall survival in our study after multivariate Cox regression analyses. However, as a proper cut-off value is important for the evaluation of the age-adjusted Charlson comorbidity index and has not met consensus in the previous studies, further research is needed [45–47].

Several other perioperative factors, including blood transfusion, opioid usage, and acute postoperative pain management strategies, may influence immunomodulation and consequently recurrence or metastases after cancer surgery. The use of opioids could have acted as a confounding factor in our study, as opioids have been found to affect tumor progression [48]. However, a previous study suggested that opioids used during oral cancer surgery are not associated with recurrence-free survival [49]. Furthermore, all patients in the current study received a constant infusion of remifentanil via a TCI pump; therefore, we presumed that there was no significant difference between the groups.

Our study suggested that advanced tumor stage was associated with poor survival and higher recurrence risk, which was consistent with the result of a previous study [50]. Older age and ASA class III were also associated with poor tumor prognosis, as observed previously [51]. A large-scale prospective study found that patients with a lower BMI had poor survival, and our results showed that a high body mass index was related to reduced death risk [52]. Poorly differentiated SCC was reported to increase the risk of recurrence, which was verified by our results [53]. A previous study has suggested that delayed reconstruction after radical mastectomy for breast cancer increases the risk of cancer recurrence [54]. Our results showed that immediate reconstruction after oral cancer surgery was not associated with cancer recurrence, despite patients usually having advanced tumor stage, similar to the previous literature [55]. The reason could be that patients who underwent flap reconstruction were likely to have more radical tumor resection [55]. In our study, packed red blood cells administered intraoperatively seemed to be associated with worse recurrence-free survival rates; however, no statistical difference was found. Due to the lack of information in the database, it was difficult to further evaluate the transfusion amounts (including the postoperative period).

Our study has some methodological limitations. First, because of the retrospective nature of this study, the results may be affected by confounding factors and selected bias. Although we used propensity score matching to balance the baseline characteristics of patients and treatment between groups, we were limited to the covariates in the medical records. Additionally, all the ASA class III patients in the original sevoflurane group were included in the matched cohort, but only 15 of the 55 patients in the original propofol group were included in the matched cohort, and this perhaps represents a selection bias. Second, because the electronic medical record system in our institution was updated in January 2014, we could only include patients from then on. Fortunately, through the calculation of sample size, the number of cases in the study was still sufficient. Furthermore, as our study data were based on 2 consecutive years, the tumor therapy during this period was basically the same. Therefore, confounding factors due to improved therapeutic approaches could be reduced. Third, perioperative factors, such as β-adrenoceptor antagonists and nonsteroidal anti-inflammatory drugs, which were reported to have an effect on cancer recurrence, were not well assessed in our study [44]. Due to the inadequacy of the electronic medical record system, it was difficult to accurately record total usage and to evaluate their effects on the results. Finally, postoperative care was not standardized at our institution. The reason for choosing postoperative care was not recorded or balanced. Prospective controlled studies are worth clarifying these issues.

In conclusion, propofol-based TIVA did not improve either overall survival or recurrence-free survival compared with sevoflurane-based anesthesia in patients undergoing oral cancer surgeries. The current study suggested that propofol-based TIVA could not provide clinical benefit compared with sevoflurane in terms of long-term oncological outcomes in oral cancer surgery.

## Supporting information

S1 Checklist STROBE statement—Checklist of items that should be included in reports of observational studies. (DOCX)
